# Non-functional Pituitary Adenomas: Analysis of Delayed Diagnosis in Mexico

**DOI:** 10.7759/cureus.45645

**Published:** 2023-09-20

**Authors:** Sergio Moreno Jiménez, Issac Vargas-Olmos, Andrea Ceballos-Arana, Karen A Miranda-Fernández, Dan Morgenstern-Kaplan, Fabiola Flores-Vázquez, Álvaro Bedoya-Gómez, Paula A Contreras-Núñez

**Affiliations:** 1 Neurological Center, American British Cowdray (ABC) Medical Center, Mexico, MEX; 2 Neurosurgery, Instituto Nacional de Neurología y Neurocirugía Manuel Velasco Suárez, Mexico, MEX; 3 Internal Medicine, American British Cowdray (ABC) Medical Center, Mexico, MEX; 4 Medicine, Universidad Anáhuac Mayab, Mérida, MEX; 5 Radiosurgery Unit, Instituto Nacional de Neurología y Neurocirugía Manuel Velasco Suárez, Mexico, MEX; 6 Internal Medicine, Jackson Memorial Hospital, Miami, USA; 7 Medicine, Universidad Anáhuac Mexico, Mexico, MEX; 8 Radiation Oncology, American British Cowdray (ABC) Medical Center, Mexico, MEX; 9 Neurosurgery, Hospital Universitario Hernando Moncaleano, Neiva, COL; 10 Radiosurgery, Instituto Nacional de Neurología y Neurocirugía Manuel Velasco Suárez, Mexico, MEX

**Keywords:** neurosurgery in developing countries, global healthcare systems, visual symptoms, early diagnosis, pituitary adenomas

## Abstract

Background: Although tumors of the central nervous system (CNS) are rare, they can cause significant morbidity and mortality. The clinical presentation of patients with non-functional pituitary adenomas (NFPA) ranges from being completely asymptomatic to causing pituitary, hypothalamic, or visual dysfunction due to their large size. Patients usually arrive with large tumors at the time of diagnosis.

Objectives: Try to describe the characteristics of NFPA and explain the causes of delayed diagnosis.

Methods: We carried out a retrospective study including 58 patients with NFPA and analyzed the tumor volume at the time of diagnosis and its relationship with sociodemographic and health sector variables.

Results: Low socioeconomic status (SES) was associated with high tumor volume (SES 1-2 of 17.4 cm^3^ vs 3-6 of 11.7 cm^3^, p=0.018), and the time between first consultation and diagnosis was longer in the public sector than in the private sector (13.5 months vs 5.1 months). The time between the first symptom and the first consultation was shorter when they had visual impairment than when they did not (4.1 vs 18.4 months, p=0.006).

Conclusions: On the one hand, citizens should be made aware that a visual deficit should make them go to a medical check-up, and on the other hand, strengthen the health system so that they have the NFPA as a differential diagnosis in patients with some visual alteration. Socioeconomic inequality in our country undoubtedly puts the underprivileged at greater risk.

## Introduction

Pituitary adenomas are the second most frequent intracranial neoplasm behind meningiomas [1]. They are often asymptomatic and incidentally diagnosed, especially non-functional pituitary adenomas (NFPAs) [2]. The clinical presentation of patients with NFPA ranges from being completely asymptomatic to causing pituitary, hypothalamic, or visual dysfunction due to their large size. From the neurological point of view, they can occur mainly with headache (19-75%), campimetric defect (79.6%), and pituitary apoplexy (7-9.5%) and, from the endocrinological point of view, with discrete elevation of serum prolactin, hypopituitarism (37-85%) or panhypopituitarism (6-29%) [3]. Current treatment consists of endoscopic or microscopic transsphenoidal resective surgery, and monitoring, reoperating, or treating with ionizing radiation in cases of residual [4]. Giant adenomas are defined as those measuring more than 40 mm in maximum diameter, corresponding to 5-14% of cases, and radical resection is achieved in less than 50% of cases with 10-20% complications [5].

Timely detection of NFPA would allow treating patients with smaller tumors and a better treatment outcome. We consider that there is a delay in the diagnosis of patients with NFPA, and the objective of this initial work is to try to describe the characteristics of NFPA and explain the delay in diagnosis [6].

## Materials and methods

We interviewed 58 patients recently diagnosed with NFPA, without previous treatment, who entered the neurosurgery service of the National Institute of Neurology and Neurosurgery (INNN) in Mexico City one day before surgery, in order to know how, at what time and who made the diagnosis; patients were recruited as they were admitted to the hospital, in a period that included from the first of March 2018 to the first of March 2019. The inclusion criteria were patients with NFPA without any previous treatment, with skull magnetic resonance imaging (MRI) with and without contrast (Gadolinium) with spoiled gradient recalled echo (SPGR) sequence, and with neuro-ophthalmological and neuro-endocrinological assessment.

We consider the following variables for the analysis: sex (man/woman), first medical contact (public/private), educational level (without studies, primary, secondary, preparatory/baccalaureate, technical career, bachelor's degree, postgraduate), dichotomized educational level (up to secondary finished/more than secondary), socioeconomic level (1-6), dichotomized socioeconomic level (1-2/3-6), presence of clinical features such as headache, impaired visual acuity, impaired visual field, asthenia, decreased libido, impotence, menstrual disturbance, and pituitary stroke. Age at diagnosis, initial symptom-consultation time in Sx-Cx months, consultation-diagnosis time in Cx-Dx months, the time between initial symptom and diagnosis in Sx-Dx months, and tumor size (cm^3^) and dichotomized tumor size (<40 mm/≥40 mm).

Statistical analysis

The categorical variables are presented as frequencies and percentages. Quantitative variables with means, standard deviation, median, and interquartile range. We performed the Shapiro-Wilk test, which showed different distributions than normal in all cases.

For the comparison of means, we used the Mann-Whitney test, considering a statistically significant difference of p<0.05. For correlation between the different variables, we used the Spearman correlation test.

## Results

Descriptive analysis

Of the total number of patients, 32 (56.2%) were women and 26 (44.8%) men. The distribution within socioeconomic levels was I 32 (55.2%), II 13 (22.4%), III 7 (12.1%), V 4 (6.9%), and VI 2 (3.4%). In relation to the level of education, nine (15.5%) had none, 14 (24.1%) were from primary school, 12 (20.7%) from secondary school, 14 (24.1%) from college, and nine (15.5%) were students. Regarding the type of health system, 27 (47.4%) of patients went to the private system, while 30 (52.6%) went to the public in the first instance. In relation to the first symptoms of presentation, 23 (51.2%) presented decreased visual acuity, 20 (34.5%) headache, and seven (12.1%) with alteration of visual fields. The main discomfort for which they went to the doctor was visual disturbance in 46 (78.9%) of the cases.

Demographic, educational, and socioeconomic status characteristics can be found in Table 1. 

**Table 1:** Baseline demographic characteristics of patients.

Demographics		
Sex	N	%
Women	32	56.2
Men	26	44.8
Socio-economic level		
1	32	55.2
2	13	22.4
3	7	12.1
4	0	0
5	4	6.9
6	2	3.4
Academic level		
None	9	15.5
Elementary school	14	24.1
High school	12	20.7
University	14	24.1
High-grade study	9	15.5
First contact health system		
Private	27	47.4
Public	30	52.6

The clinical characteristics of the patients are shown in Table 2. 

**Table 2:** Clinical characteristics of patients with NFPA. NFPA: non-functional pituitary adenomas, VA: visual acuity, VF: visual fields.

Initial symptom	N	%
Decreased VA	23	39.7
Headache	20	34.5
VF alteration	7	12.1
Galactorrhea	3	5.2
Hypopituitarism	1	1.7
Asthenia	1	1.7
Other	3	5.2
Symptom that motivated to go to the consultation	N	%
Decreased VA	30	51.2
VF alteration	16	27.6
Headache	5	8.6
Galactorrhea	4	6.9
Menstrual disturbance	1	1.7
Symptoms present at any time of the disease	N	%
Decreased VA	52	89.7
Headache	43	74.1
VF alteration	34	58.6
Asthenia	18	31.0
Menstrual disturbance	7	12.1
Decreased libido	7	12.1
Pituitary stroke	5	8.6
Galactorrhea	3	5.2
Hypopituitarism	5	8.6
Impotence	2	3.5

The descriptive analysis of the quantitative variables is shown in Table 3. 

**Table 3:** Descriptive characteristics of quantitative variables. Sx: symptom, Cx: consultation, Dx: diagnosis, X: lateromedial, Y: anteroposterior, Z: dorsoventral, RIC: interquartile range; Md: medium, Inf. L: inferior limit, Sup. L: superior limit, IQR: interquartile range, SD: standard deviation, CI: confidence interval.

Variable							95% CI	
	Min	Max	Mean	SD	Md	IQR	Inf. L.	Sup. L.
Age	24	77	49.3	12.3	50	15.8	46.0	52.5
Sx-Cx time	0	84	11.0	19.1	3.0	10.0	5.8	16.1
Cx-Dx time	0	53	9.6	12.6	4.0	10.0	6.3	13.0
Sx-Dx time	1	87	20.6	22.4	11	21.8	14.7	26.5
No. consultations	1	12	4.0	1.7	4.0	2.0	3.6	4.5
X	16.7	63.0	29.8	7.9	27.7	7.8	27.7	31.9
Y	11.7	71.5	34.3	8.9	33.9	9.7	31.9	36.7
Z	13.7	70.7	27.7	8.5	26.4	7.9	25.4	29.9
Volume (cm^3^)	2.6	55.5	16.1	10.0	14.0	9.1	13.5	18.8

Inferential analysis

Correlation Analysis

We performed Shapiro-Wilk normality test to the variables time symptomatology-consultation (Sx-Cx), time between consultation and diagnosis (Cx-Dx) and time between symptomatology and diagnosis (Sx-Dx), as well as tumor volume, finding that the four have different distribution than normal (W=0.810, p=0.0001; W=0.810, p=0.0001; W=0.776, p=0.0001; and W=0.810, p=0.0001), respectively. All times are expressed in months while the volume is always expressed in cm^3^.

In order to analyze the strength of the relationship between the time elapsed between the Sx-Cx time and the tumor volume at the time of diagnosis, we performed a Spearman correlation analysis, finding an r=0.095 (p=0.47), while for the Cx-Dx time against the tumor volume, it was r=0.192 (p=0.148). The result for the time between time Sx-Dx was (r=0.204, p=0.125). None showed a statistically significant correlation.

Socioeconomic Status, Volume, and Time

As for the SES, grouping levels 1-2 and 3-6, we found a tumor volume of 17.4 (SD=10.8) and 11.7 (SD=5.3), respectively, with a value of p=0.018. When analyzing the SES against time, Sx-Cx, Cx-Dx, and Sx-Dx, we found results in months of 12.0 (SD=21.6) vs 7.3 (SD=9.0) (p=0.49), 10.4 (SD=13.3) vs 6.8 (SD=10.3) (p=0.13), and 22.5 (SD=24.7) vs 14.1 (SD=26.8) (p=0.33), respectively.

Levels of Study, Volume, and Time

By dichotomizing the maximum degree of studies from "no studies" to complete secondary in the first group, and complete preparatory onwards in the second, we found a tumor volume of 15.2 (SD=7.3) and 17.5 (SD=13.3) (p=0.49), respectively. In relation to the time Sx-Cx, Cx-Dx, and Sx-Dx, we found 8.5 months (SD=15.6) vs 14.8 (SD=24.2) (p=0.38), 10.0 (SD=14.0) vs 9.0 (SD=10.8) (p=0.3), and 18.5 (SD=19.4) vs 23.8 (SD=26.8) (0.66), respectively.

Type of Health System, Volume, and Time

There was no difference between going to a public vs private clinic and tumor volume being 16.1 (SD=9.3) and 16.1 (SD=11.1) (p=0.41). Regarding the type of office and the Sx-Cx, Cx-Dx, and Sx-Dx times, we found 14.1 months (SD=22.8) vs 7.4 (SD=14.3) (p=0.07), 13.5 (SD=15.7) vs 5.1 (SD=5.5) (p=0.037), and 27.6 (SD=25.3) vs 12.5 (SD=16.0) (0.004), respectively.

Initial Symptom, Volume, and Time

The relationship between the presentation of the clinical picture with headache and tumor volume is found to be 16.9 (SD=11.1) with headache and 15.6 (SD=9.5) without headache (p=0.43). Regarding the relationship between the presence of headache as an initial symptom and the times Sx-Cx, Cx-Dx, and Sx-Dx were 16.5 months (SD=25.5) vs 7.3 (SD=13.4) (p=0.036), 6.6 (SD=6.5) vs 11.6 (SD=15.3) (p=0.69), and 23.1 (SD=26.7) vs 19.0 (SD=19.7) (p=0.35). The relationship between the presence of visual impairment as an initial symptom (visual acuity or visual field) and tumor volume was 17.1 (SD=9.8) vs 15.1 (SD=10.5) (p=0.065). In relation to the times Sx-Cx, Cx-Dx, and Sx-Dx were 4.1 (SD=5.8) vs 18.4 (SD=25.6) (p=0.006), 11.9 (SD=14.1) vs 7.1 (SD=10.7) (p=0.088), and 16.0 (SD=14.4) vs 25.5 (SD=28.4) (p=0.76), respectively.

Prediction Analysis of a Giant Tumor

**Figure 1:**
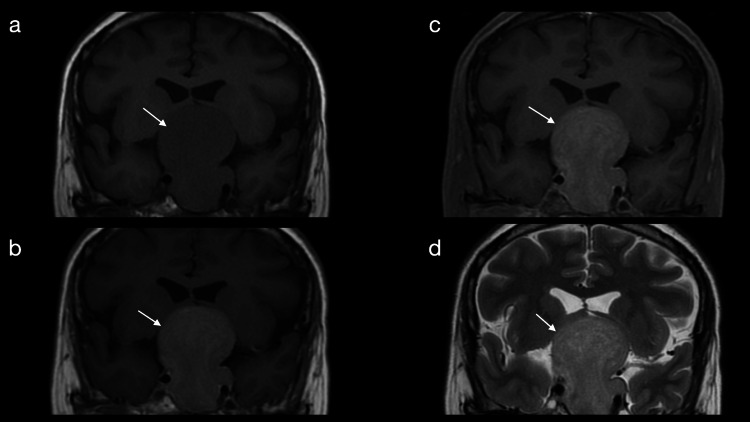
An MRI of the skull with a non-functional pituitary macroadenoma is observed in coronal cuts in sequences (a) simple T1, (b) contrasted T1, (c) T1 contrasted with fat suppression, and (d) T2. MRI: magnetic resonance imaging.

The SES divided into levels 1-2 and 3-6 was significantly associated with the presence of a giant tumor defined as greater than or equal to 40 mm (x^2^=3.92, p=0.048) (Figure 1).

No differences were found in the following variables: sex (x^2^=0.002, p=0.96) and first contact (x^2^=0.35, p=0.56), (x^2^=0.19, p=0.66).

## Discussion

Patients at the INNN are classified according to their socioeconomic status, considering the following variables: family income, occupation, family discharges, housing, and family health. Each of these variables is assigned a score according to the patient's situation and is classified into levels from 1 to 6. Most of our patients belong to the low socioeconomic classes, being 77.6% level 1 or 2, and only 22.4% belong to the more favored socioeconomic classes.

The most frequent initial symptom was visual impairment, defined as the sum of alteration of visual acuity and visual field, at 51.8%, followed by headache at 34.5%. The leading cause that motivated the patient to go to the first consultation was the visual alteration, which was 78.8%. There are reports of some pituitary deficiency in up to 60-85% of patients with non-functional pituitary macroadenomas; however, the vast majority of these patients are asymptomatic from the neuroendocrinological point of view, so they are not associated with the initial symptoms [7]. The leading clinical feature at presentation, according to several reports, is a mass effect that causes visual disturbance, headache, or hypopituitarism [8,9].

All patients had visual impairment at some point in the clinical course of the disease, while 74.1% presented headache, which could not always be attributed to the presence of an adenoma.

The time elapsed between the first symptom and the first consultation could be a time mainly attributable to the patient, while the time between the first consultation and the etiological diagnosis can be mainly attributable to the health system; the first was 11.0 months and the second was 9.6 months. The average number of consultations between the first consultation and the diagnosis was four consultations. The delay times have been previously categorized in the Anderson Model of Total Patient Delay [10]. We adapted part of this model to our research.

There are works in the literature regarding the symptomatology of presentation of cancer patients, socioeconomic deprivation, reporting that delay on the part of the patient is defined as the time that elapses from the discovery of the first symptom until they go to the health professional, and is responsible for the highest proportion of delays on the way to start treatment [11].

Thirty (52.6%) patients had first contact with the public health system and 27 (47.4%) with a private hospital. Mexico has the highest rate among private hospitals and public hospitals, being 28.6 private hospitals per 11.4 public hospitals per million inhabitants [9], so it is not uncommon that, despite being patients with low economic resources, almost half have gone to private medical service at the beginning.

We found no correlation between the time elapsed between the first symptom and the first consultation or the time between the first consultation and the diagnosis with the volume of the NFPA.

We found a statistically significant difference between the socioeconomic status of our patients and tumor volume at the time of diagnosis. In the first group of NSE 1-2, the volume was 17.4 cm^3^, while in the second group of SES 3-6, it was 11.7 cm^3^ (p=0.018). That is, patients belonging to more favored socioeconomic levels were diagnosed with a lower tumor volume. This can be explained by patients belonging to upper socioeconomic classes could have greater accessibility to health services, to the necessary studies, and therefore to a more timely diagnosis. The time between the patient's first symptom and the first consultation was 12 months in the SES 1-2 group and 7.3 months in the SES 3-6 group; however, we did not find the difference statistically significant. It is also described in the literature that people who belong to less favored socioeconomic levels have a lower survival rate in diseases such as cancer; this is due to the late diagnosis of the disease, although the mechanisms underlying this cause have not yet been fully understood [12].

The educational level of 60.3% of our study participants was not more than the secondary level. However, we found no significant relationship in terms of the degree of study of our patients and the tumor volume they presented, nor with the times studied.

Regarding the type of health system to which patients went for the first time, it was 30 (52.6%) in the public sector and 27 (47.4) in the private sector. The volume of the tumor at the time of diagnosis was practically the same in both groups (16.1 cm^3^). However, we did find statistically significant differences between the time elapsed between the first consultation and the diagnosis, which was 13.5 months when it was in the public sector and 5.1 months in the private sector (p=0.037). Considering the total time between the first symptom and diagnosis was 27.6 months in the public and 12.5 months in the private (p=0.004).

There is a significant difference in terms of the time in months that elapses since patients begin with the symptoms to which the diagnosis is made, depending on whether they go to a public or private office in the first instance. We find that times are shortened and, therefore, a timely diagnosis is reached earlier if the first contact of patients with the health system is the private medium. This can be explained by the resources that are available in the private environment, the greater accessibility to diagnostic equipment, and above all, the time factor that here plays an important role since the patient does not have to wait so long to be able to schedule an appointment with the specialist or for the realization of paraclinical studies. Although, of course, it could be explained because patients who come to a private practice have more resources. The time between the first consultation and the diagnosis is mainly attributable to the health system.

When the initial symptom was headache, there were no differences in tumor volume; however, there was a difference in that the time between the headache and the first consultation was greater in the presence of headache, contrary to what we might have suspected (16.5 months vs 7.3 months, respectively, p=0.036). This finding is difficult to interpret; however, one explanation could be that headache, being a universal symptom, usually loses weight in terms of functioning as an alarm symptom, especially since the headache associated with an NFPA is not usually of great intensity.

Regarding the relationship between tumor volume and the presence or absence of visual alterations as an initial symptom, no difference was found. We would have expected to find that when the patient has visual alterations, he would arrive with a lower tumor volume for a more timely diagnosis.

We found that in patients who presented visual alterations as an initial symptom, the time that elapsed from the onset of symptoms to consultation with the doctor was less than in patients who did not present visual alterations, being 4.1 months vs 18.4 months (p=0.006). These results are in accordance with what is reported in the literature since NFPA does not produce clinical symptoms derived from hormonal secretion, and this may contribute to a delay in diagnosis, so most patients seek medical attention for signs and symptoms due to the mass effect, such as visual alterations and headache [13].

The number of giant NFPA we had was 11 (19%). It has been found in works on breast cancer a statistically significant relationship between the time of delay diagnosis and the clinical tumor size, so that the greater the diagnostic delay, the greater the tumors that are found. And as tumor size increases, the chances of survival decrease [14]. We found no reports of benign tumors. In a series of 118 patients with NFPA, with a maximum diameter of 4 cm, they divided their patients into grade I, those located within the Turkish sella, below the sellar diaphragm and without invading the cavernous sinus; grade II were the ones that invaded the cavernous sinus; grade III, the giant adenomas that raise the floor of the cavernous sinus; and grade IV, those that transgressed the sellar diaphragm or the roof of the cavernous sinus and invaded the subarachnoid space. Grade IV tumors had large residuals [15]. Although NFPAs are benign tumors, tumor volume influences the patient's prognosis since as the lesion grows, it compresses adjacent structures, making their resection more difficult.

It will be important to carry out studies considering the impact that a delay in diagnosis has not only on the signs and symptoms of the patient but on other psychosocial elements such as cognitive function, sexuality, psychological well-being, social functioning, behavior, and perception of the disease among others, as described in the "Wilson-Cleary" quality of life model (HR-QoL) [16].

The main limitations of the study are the small sample size, in addition to being a retrospective study with the biases that this implies, especially memory bias.

## Conclusions

A timely diagnosis of NFPA is challenging. On the one hand, citizens should be made aware that a visual deficit should make them go to a medical check-up, and on the other hand, strengthen the health system so that they have the NFPA as a differential diagnosis in patients with some visual alteration. We found that low socioeconomic status is associated with a delay in going to the doctor and a larger size of the tumor at the time of diagnosis, more than the degree of studies. Socioeconomic inequality in our country undoubtedly puts at greater risk those who have fewer resources.
